# Reflections on the discovery GLP-1 as a satiety hormone: Implications for obesity therapy and future directions

**DOI:** 10.1038/s41430-024-01460-6

**Published:** 2024-06-18

**Authors:** Arne Astrup

**Affiliations:** Department of Obesity and Nutritional Sciences, Novo Nordisk Foundation, Hellerup, Denmark

**Keywords:** Obesity, Feeding behaviour

## Abstract

Scientists were chasing an incretin hormone, and when GLP-1 was finally discovered, we found that it had a pronounced satiety effect, slowed down gastric emptying, and actually reduced postprandial insulin response. These mechanisms are the basis for the highly efficacious GLP-1 analogues that today offer safe and effective treatment in millions of people living with obesity. Moreover, the combined GLP-1 mechanisms of weight loss and delayed carbohydrate absorption may also be the key drivers of remission of type 2 diabetes and reduced cardiovascular events found by GLP-1 analogues.

## Development of a rigorous methodology to measure appetite and food intake in humans

**Fig. 1:**
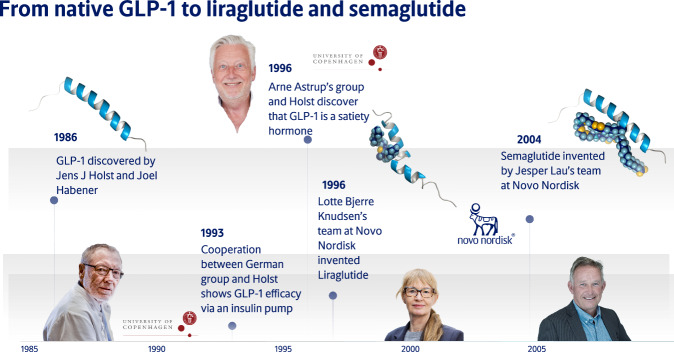
Time line showing the discovery of GLP-1 i 1986, the satiety hormone effect i 1996, and the synthesis of the long-acting analogues liraglutide and semaglutide, and the key persons involved.

With the discovery of GLP-1 in 1986 simultaneously by Jens Juul Holst and Joel Habener a cascade of important physiological discoveries followed [1] (Fig. 1). Their identification of the amino acid sequence of the biologically active GLP-1 hormone laid the groundwork for drugs in the management of type 2 diabetes. Gut peptide hormones such as GLP-1 could potentially also have an impact on appetite regulation by effects on gastric emptying and direct CNS effects, but we needed a robust methodology that could be used to measure acute effect of putative hormones on subjective appetite and spontaneous food intake. We^*^ developed a study design using visual analogue scales (VAS) for measurement of subjective appetite recordings during a fixed breakfast meal test, and combined it with a subsequent *ad libitum* lunch meal test that measured consumed energy. The putative satiety effect of a compound would be seen as an effect on satiety VAS during the breakfast meal and on the spontaneous caloric intake at the lunch meal. VAS had been mainly used to measure subjective sensations (e.g. pain) and studies of appetite lacked validated measures. In an initial pilot study, we demonstrated the need for more comprehensive validation [2].

Together with John Blundell, we developed the protocol: on two different test days we examined 55 healthy individuals and recorded their appetite sensations before breakfast and every 30 min during the 4.5 hr postprandial period under the same conditions [3]. We also examined if diet standardization the days prior to the test days influenced the results. The study showed the validity of quantification of hunger, satiety, and prospective food consumption measured by VAS as well as measurement of *ad libitum* energy consumption and showed that the appetite recordings during the breakfast meal predicted the energy intake during the lunch meal [3]. These results provided us with the essential methodology to design the GLP-1 infusion study and provided information on the estimated number of participants needed for adequate statistical power. This VAS paper is today a classic methods paper in this field [3] and has been cited 2,382 times.

^*^In this article “we” is used as a reference to the group of scientists, dieticians, and technical staff in my department who contributed to own studies cited, and the key collaborating scientists from other labs including Jens Juul Holst and John Blundell. Contributors from my own department are mentioned in the Acknowledgment.

## Discovery of GLP-1 as a satiety hormone

In 1994 I took up the idea with Holst that GLP-1 could also be a mediator of meal-induced satiety, and we designed a study to infuse GLP-1 into normal human volunteers. Meanwhile, in January 1996 the first rodent study suggested that GLP-1 could be a central regulator of feeding behavior and satiety based on intracerebroventricular injection studies [4], but soon thereafter the results of other rodent studies using peripherally injected GLP-1 argued against an appetite effect [5].

**Fig. 2:**
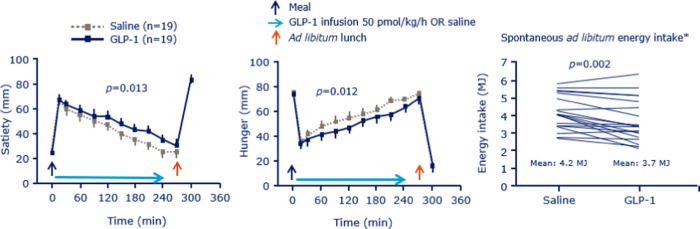
Effects of GLP-1 infusion versus placebo on appetite recordings following a fixed breakfast meal, and on spontaneous energy intake during an *ad libitum* lunch meal in 20 healthy individuals [6]. The pivotal findings of the study in which we infused GLP-1 in human subjects showed that it enhanced satiety reduced hunger during a fixed breakfast meal and reduced spontaneous energy intake by 12% during an ad libitum lunch meal [6].

The study was conducted in 1995–1996, and we used commercially available synthetic, human GLP-1 (7–36 amide) [6]. We conducted the trial in 20 young, healthy, normal-weight men in a placebo-controlled, randomized, blinded, crossover design. Infusion of GLP-1 or saline was initiated simultaneously with the consumption of the breakfast test meal. VAS were used to assess appetite sensations, and at lunchtime an ad libitum test meal was offered. We found that, after the energy-fixed breakfast, GLP-1 markedly enhanced satiety and fullness compared with placebo (Fig. 2) [6]. Furthermore, spontaneous energy intake at the ad libitum lunch was reduced 12% by GLP-1 infusion (Fig. 2). We were quite excited by the findings, as this study was the first to demonstrate a physiological role of GLP-1 in appetite control and energy intake in humans.

An invention was submitted for a patent on 12 November 1996 [7], and the scientific article was submitted for publication in the *Journal of Clinical Investigation* 20 June 1997 and published 1st February 1998 [6]. The experimental set-up and execution of the studio are reproduced in a short video with actors (https://www.youtube.com/watch?v=4vbsEpC7Hsw).

**Fig. 3:**
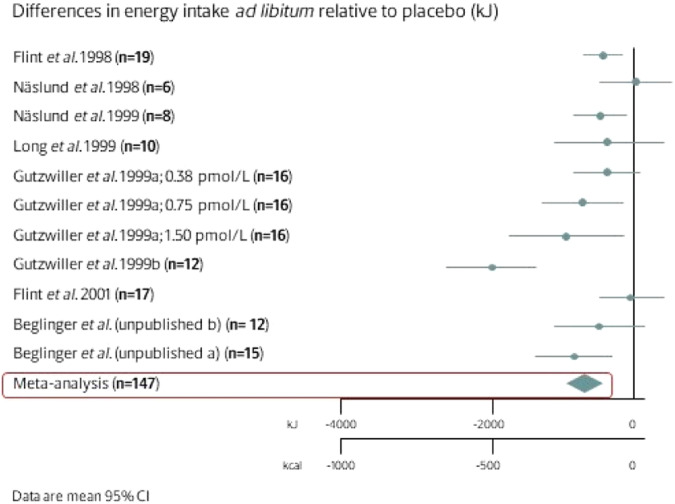
Meta-analysis of the effect of GLP-1 infusion versus saline on energy intake in human individuals. Data obtained from *n* = 147 volunteers following infusions with GLP-1 or saline. Adopted from [11].

In September 1998, Näslund et al. failed to detect an effect of GLP1 on food intake [8]. However, that study was done in only 6 subjects, raising concerns about the lack of statistical power to detect a clinically relevant effect. In 1999, Gutzwiller et al. showed a dose-dependent reduction in food intake with GLP-1 infusion in 16 healthy male subjects [9]. Subsequently, we showed that GLP-1 induces satiety equally well in people with obesity [10]. These effects could be at least partially attributed to the slowing of gastric emptying. By 2000, seven studies had been published on the acute effect of peripheral GLP-1 administration on ad libitum energy intake. Four found energy intake to be significantly reduced, whereas the remaining studies failed to find an effect – again potentially due to limited statistical power and also low GLP-1 infusion rates. We therefore conducted an individual participant data analysis, pooling raw data from the existing studies [11]. Six infusion studies provided us with 147 participants, and we found that GLP-1 reduced energy intake dose-dependently in both lean and overweight participants (a mean of 13%, *P* < 0.001, Fig. 3) [11]. There was also a dose-response effect on the reduction in emptying rate.

In close collaboration with Jens Juul Holst, we conducted a series of infusion studies on the satiety effects of other gut hormones including GLP-2 and PYY. Beyond contributing to the conceptual knowledge of appetite and energy balance regulation in humans [12], we also made potentially clinically important discoveries, such as why dietary protein provides greater immediate satiety than the other macronutrients and why milk is more satiating than sugar-sweetened soft drinks (both dietary exposures increase GLP-1 relative to their comparators) [13].

## From physiology to pharmacology

Our discovery of GLP-1 as a satiety hormone was patented in collaboration with the pharmaceutical company Novo Nordisk A/S in November 1996 [7] (I receive no royalty or other payments). Mads Krogsgaard Thomsen, at the time R&D executive in the Diabetes Care Division of Novo Nordisk, undertook the initiative to synthesize long-acting GLP-1 analogues (Fig. 1), and the first GLP-1 analogue brought into clinical development was liraglutide, a once-daily injection. I led a phase II dose-ranging trial on liraglutide, and we found that over 20 weeks the 3.0 mg/d dose had an optimal profile of effectiveness and tolerability [14]. The trial, extended for 2 years, found that completers maintained a 7.8 kg weight loss from baseline [15]. In the phase III liraglutide program (SCALE study) ~4000 participants with obesity were studied for up to three years [16].

To date, liraglutide, and the more effective once-weekly semaglutide, have been approved in the US and Europe for the treatment of obesity, with unprecedented weight loss of up to 15–20%. Presently, 19 different GLP-1 analogues are in development for the treatment of obesity by almost as many companies. In the last three months of 2023, more than 9 million people were treated by GLP-1 analogues in the US, with anticipation of continued rapid growth in usage.

## The importance of GLP-1 therapies for type 2 diabetes and CVD

Today it is well established that excessive body fat together with lack of regular physical activity, together with a genetic predisposition, are the main causes of type 2 diabetes, contributing as well to a substantial proportion of cardiovascular diseases, cancers, and other chronic degenerative diseases. A large body of evidence has demonstrated that complete remission of type 2 diabetes can be achieved by a major weight loss. Until recently, the only effective treatment for obesity was bariatric surgery, and a century of drug development for obesity can be essentially described as a failure until now. GLP-1 analogues have completely changed this bleak therapeutic picture. Moreover, GLP-1 analogues cause a reduction in cardiovascular events in non-diabetic individuals [17]. The mechanisms responsible for these effects are not entirely clear, but the slowing of gastric emptying may be the key driver.

## How do GLP-1 analogs confer health benefits?

**Fig. 4:**
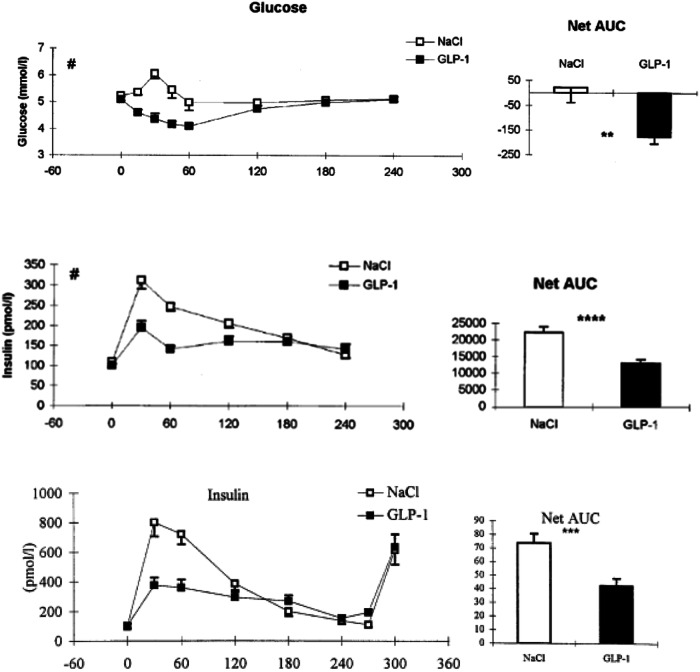
The plasma glucose and insulin response to a mixed meal during infusion with either GLP-1 or saline are shown in normal-weight individuals (upper panel) and in individuals with obesity (lower panel), respectively. The quantitative reduction in insulin response amounts to 40%. The reduced glucose and insulin responses are consistent with the marked reduction in gastric emptying causing delayed absorption of glucose and hence a lower glycemic index of the meal (from refs [6, 10]).

An apparent paradox is that whereas GLP-1 increases glucose-stimulated insulin secretion in the isolated pancreas – fulfilling the definition of an incretin – but it lowers insulin secretion in physiologically relevant conditions [6, 10, 18]. In our discovery studies GLP-1 enhanced satiety during a meal but lacked the insulinotropic effect in the natural setting of people consuming mixed meals (Fig. 4).

**Fig. 5:**
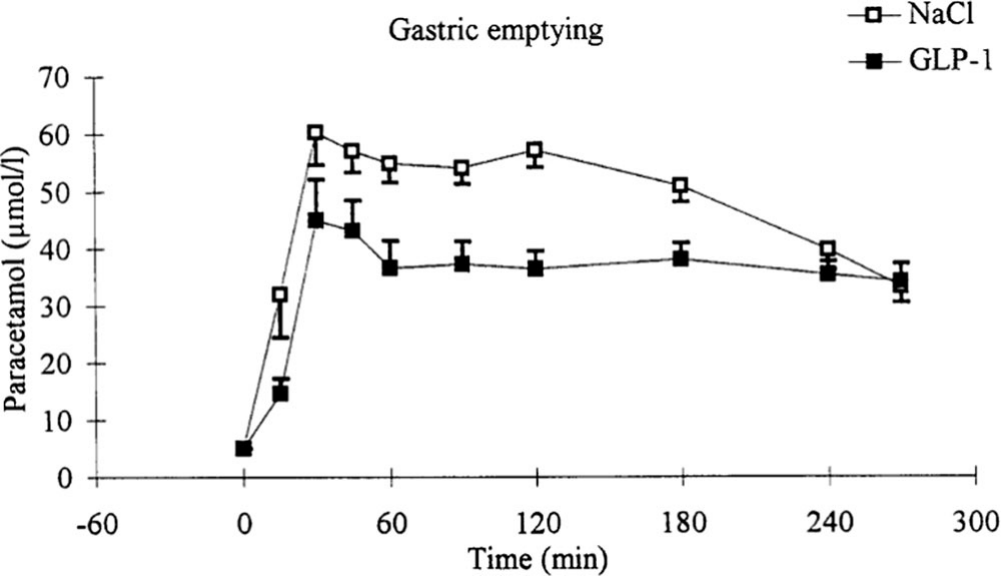
GLP-1 inhibits gastric emptying in human individuals as shown by the retarded absorption of ingested paracetamol [10].

We found this paradox to be explained by GLP-1 delaying gastric emptying and the rate of carbohydrate absorption after a meal, biological actions that dominant direct effects on insulin secretion at the B-cell, both in both normal-weight individuals and in non-diabetic individuals with obesity [6, 10] (Fig. 5). Despite all major review articles highlighting GLP-1 as an incretin hormone [18], meal-test studies and studies using GLP-1 analogues actually show the opposite.

## More satiety hormone than incretin

From this perspective, GLP-1 is more appropriately understood as a satiety hormone, rather than an incretin. It was also an early observation that a higher dose of GLP-1 is needed to produce satiety and reduced food intake than to the antidiabetic effect, so a low dose GLP-1 analog could be used to study weight loss independent effects of GLP-1. In an 8-week study using low-dose liraglutide (0.6 mg/day) in patients with type 2 diabetes no weight or fat loss was produced, but both fasting and postprandial glucose were substantially reduced, but neither fasting nor postprandial insulin (or C-peptide) response was increased [19]. This supports that the short-term anti-diabetic effect of GLP-1 is mainly due to slowing of gastric emptying and consequently a reduced rate of carbohydrate absorption. The most common side effects of GLP-1 analogues are nausea and vomiting, which have been attributed to delayed gastric emptying [14–16]. The reports of increased residual gastric content after long-term treatment with GLP-1 analogues emphasize that the effect on gastric emptying is sustained, even though that it does normally not pose a risk for postoperative respiratory complications [20].

## Reductions of glycemic index and hyperinsulinemia by GLP-1

**Fig. 6:**
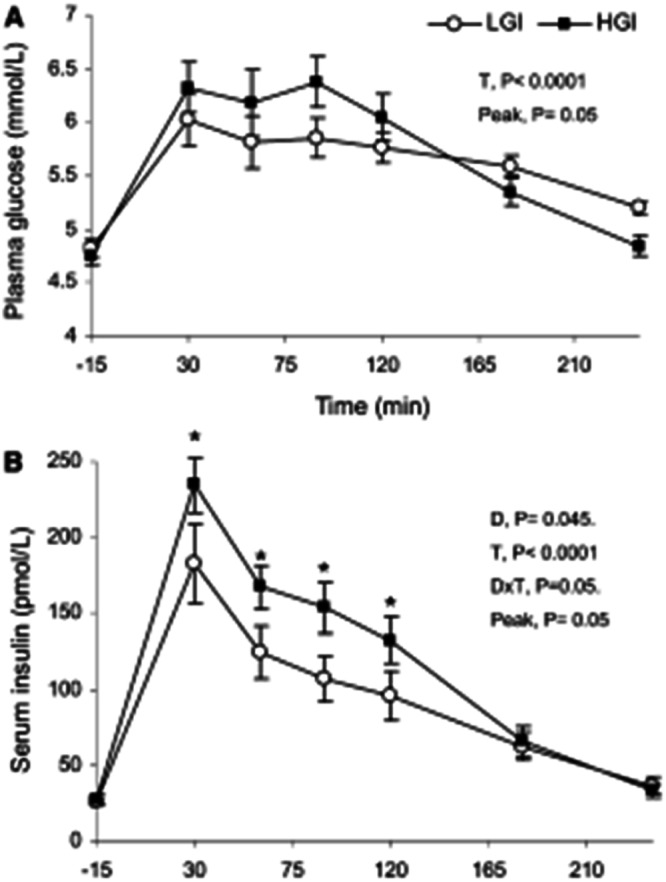
The effect of delaying carbohydrate absorption on glucose and insulin responses. The difference between a meal with high versus low glycemic index (GI) on plasma glucose (panel **A**) and insulin response (panel **B**). The difference is very similar to the lowering effect of GLP-1 on GI, and this effect is likely to contribute to the weight-loss-independent effect of GLP-1 analogues on type 2 diabetes and cardiovascular disease in analogy with the effects of a low GI diet [21].

GLP-1 exerts an important effect directly on the brain, and this effect is probably even more important for the analogues than for the endogenous GLP-1 released after ingestion of protein and fat. My view is that the pronounced effect of GLP-1 analogues on gastric emptying is not only an important mediator of satiety and reduced food intake but also a likely mechanism responsible for its effect on type 2 diabetes and cardiovascular disease. The reduced glycemic load of meals caused by GLP-1 is very similar to that seen when replacing a meal with a high glycemic index (GI) with that of a low GI [21] (See Fig. 6). Due to the reduced food intake total carbohydrate ingestion is also reduced, which will further reduce glycemia.

Consequently, on top of the beneficial effect of weight loss caused by GLP-1 analogues these drugs also cause a substantial reduction of the glycemic load (GL = carbohydrate intake X Glycemic index), which per se reduces hyperinsulinemia and causes weight loss [22], reduce diabetes risk [23], inflammation [24], and cardiovascular risk [25]. Indeed, it is well documented that regular consumption of high-glycemic index meals, compared to isoenergetic meals with the same carbohydrate content, results in higher average 24-h blood glucose and insulin levels, higher C-peptide U-excretion, and higher HgbA1c in both non-diabetic and diabetic individuals [26, 27]. When it comes to the effect of GLP-1 analogues the reduction in postprandial glucose response, and insulinemia may actually contribute to weight loss, but also be the key drivers to the anti-diabetic effect and reduction in cardiovascular disease risks. Studies with treatment with GLP-1 analogues in type 2 diabetes have found that the initial postprandial insulin is increased after treatment, and this has been misinterpreted as evidence for a GLP-1 mediated insulin stimulation [28]. However, this phenomenon is rather a restoration of the first-phase insulin response due to the concomitant weight loss of typically 5–6 kg. The restoration of first-phase insulin response is a well-established observation caused by weight loss in type 2 diabetes [29]. The overall reduction in insulin secretion is also shown by reduced levels of C-peptide in the same studies. So GLP-1 therapy reduces insulin secretion and levels, which have implications for the understanding of the mechanisms behind its effect on weight loss.

Endogenous hyperinsulinemia may cause weight gain and obesity [30], and treatment with insulin causes substantial weight gain, and may even increase cardiovascular risk [31]. Whereas general obesity and central adiposity, in particular, are the primary factors responsible for the development of insulin resistance, postprandial increases in insulin secretion causes a further expansion of the adipose tissue mass, which further worsens insulin resistance. According to this scenario, obesity and hyperinsulinemia initiate a vicious metabolic cycle leading to deterioration of glucose tolerance and eventual development of T2DM. So, reducing hyperinsulinemia by GLP-1 analogies will contribute to weight loss and improve insulin sensitivity, and glycemic control in type 2 diabetes. This novel view on GLP-1 mechanisms has recently been highlighted by Ludwig and Holst [32].

## Perspectives

The pivotal discovery of GLP-1 was followed by the discovery of GLP-1 being a satiety hormone in humans acting on the brain and exerting a pronounced slowing of gastric emptying leading to weight loss. The combination of reduced carbohydrate intake and slowing of glucose absorption also reduces postprandial glycemia and insulinemia and may be the dominant weight-loss-independent effect on type 2 diabetes and cardiovascular disease. These findings provided a better understanding of the physiology of GLP-1 and the pharmacology of its analogues and have revolutionized the management of obesity and several comorbidities. For the first time in the history of obesity medications we see weight loss efficacy of a magnitude that might raise concern about excessive loss of lean body tissue due to the initial pronounced reduction in energy intake and subsequent risk of protein deficiency.

The effects on the brain of GLP-1 analogues go far beyond satiety but may also change food preferences in a healthier direction, but also reduce a person’s drive for alcohol, tobacco and even opioids. Consequently, the potential for this class of drugs is enormous and may be a paradigm shift not only for the management of obesity but also for several other conditions.
